# Effects of a high-fat diet on superoxide anion generation and membrane fluidity in liver mitochondria in rats

**DOI:** 10.1186/s12970-018-0217-z

**Published:** 2018-03-14

**Authors:** M Togo, N Konari, M Tsukamoto, R Kimoto, T Yamaguchi, H Takeda, I Kambayashi

**Affiliations:** 1 Graduate School of Dairy Sciences, Rakuno Gakuen University, 582, Midorimachi Bunkyodai, Ebetsu, Hokkaido 069-8501 Japan; 2 Graduate School of Medicine, Sapporo Medical University, S1 W17, Chuo-ku, Sapporo, Hokkaido 060-8556 Japan; 3 School of International Culture Relations, Tokai University, 5-1, Minaminosawa, Minami-ku, Sapporo, Hokkaido 005-8601 Japan; 4 Asahikawa National Institute of Technology, 2-2-1-6, Syunkodai, Asahikawa, Hokkaido 071-8142 Japan; 5 School of Health Sciences, Sapporo Medical University, S1 W17, Chuo-ku, Sapporo, Hokkaido 060-8556 Japan; 6 Department of Education, Hokkaido University of Education Sapporo, 3- 5, Ainosato Kita-ku, Sapporo, Hokkaido 002-8502 Japan

**Keywords:** High-fat diet, Liver mitochondria, Superoxide anion, Adrenaline method, Membrane fluidity, Spin labels

## Abstract

**Background:**

Obesity is a primary factor of lifestyle-related diseases, and the age of its onset has decreased. The reactive oxygen species (ROS), the superoxide anion, is generated in the mitochondrial electron transport chain and the damage it induces in cells may be a contributing factor to obesity-related lifestyle diseases. In the present study, the influence of the ingestion of a high-fat diet (HFD) on superoxide anion generation in rat liver mitochondria (Mt) and membrane fluidity was investigated.

**Methods:**

Male Wistar rats were fed a normal diet (ND, *n* = 6) or HFD (*n* = 6). Liver Mt were isolated and oxygen consumption, superoxide anion production (the adrenaline method), and membrane fluidity (the spin label method) were measured.

**Results:**

After 11 weeks, body weights and abdominal circumferences were higher in the HFD group than in the ND group. Mt oxygen consumption was higher in the HFD group than in the ND group. Superoxide anion production was significantly lower in the HFD group than in the ND group, while no significant changes were observed in membrane fluidity.

**Conclusion:**

Although rats developed diet-induced obesity, it did not reach the level of disease development. The promotion of lipid metabolism appeared to reduce superoxide anion production, but did not influence membrane fluidity. While superoxide anion damages cells as an oxidative stress, ROS and superoxide dismutase are essential signaling molecules in the body. The present results suggest that the continuous ingestion of a HFD impairs Mt and induces disease development.

## Background

Obesity is a growing health issue worldwide and the age of its onset has been decreasing. Since obesity progresses to diabetes, hypertension, and dyslipidemia [1], its treatment is important for the prevention of disease development. Diseases develop from obesity through a number of mechanisms, including oxidative stress induced by reactive oxygen species (ROS) [2].

An obesity-induced increase in leptin levels in the body has been shown to promote inflammatory reactions [3]. These inflammatory reactions increase TNF-α and IL-6 levels, which, in turn, activate NADPH oxidase and induce the extravascular migration of immune cells, thereby increasing ROS production and oxidizing lipids and proteins [4]. An increase in oxidative stress marker levels has been reported in obese individuals [5]. The urinary level of 8-epi-PGF2α, which is a lipid oxidative stress marker, has been correlated with obesity and becomes elevated with increases in BMI [6]. In rats fed a high-fat diet (HFD) that increased body weight, an increase in oxidative stress marker levels was detected in the liver [7] and skeletal muscle [8].

ROS are essential for normal physiological functions, gene expression, cell growth, defense against infections, and the control of vascular endothelial cells [9–11]. However, when ROS production exceeds scavenging abilities, cells are exposed to oxidative stress and are damaged [2]. Mitochondria (Mt), which consume approximately 90% of intracellular oxygen [12], constantly produce a specific amount of the superoxide anion (O·_2_^−^) in the electron transport chain in the inner membrane [13, 14]. A previous study reported that hydrogen peroxide production was increased in skeletal muscle Mt in obese rats [8]. Therefore, the influence of obesity on O·_2_^−^ production in Mt and the importance of evaluating inflammatory progression-induced ROS production need to be clarified; however, only a few studies have been conducted, and O·_2_^−^ production as an index has not yet been investigated. Another area that warrants further study is the impact of membrane fluidity and O·_2_^−^ production in relation to the lipid composition of a diet. The electron transport chain is present in the inner membrane of Mt, and membrane fluidity influences electron leakage in the inner membrane, which alters the production of O·_2_^−^. The Mt membrane is affected by the amount and components of ingested lipids and, thus, membrane fluidity is altered [15–17].

The present study had the following objectives: 1) to quantitate and evaluate Mt O·_2_^−^ production in the livers of rats with HFD-induced obesity, and 2) to clarify the relationship between Mt membrane fluidity and O·_2_^−^ production.

## Methods

### Experimental animals

Twelve male Wistar rats were used as experimental animals in the present study. Animals were maintained at a room temperature of 24 ± 2°C and humidity of 50 ± 5% under a lighting cycle (lights on: 7:00–19:00, lights off 20:00–6:00). They were housed in plastic cages at 2–3 animals/cage (width: 206 mm, depth: 365 mm, height: 197 mm), and given free access to food and drinking water. Animals only moved inside the cage throughout the maintenance period.

### Feed

Rats were given a normal diet (ND) or HFD. MF pellets (Oriental Yeast Co., Ltd.) were used for the ND, and 58Y1 with a lard content of 60% (PMI) was used for the HFD. The nutritive values (PFC ratio) of the ND were: 360 kcal/100 g; protein, 23.6 g (26.2%); lipids, 5.3 g (13.3%); and carbohydrates, 54.4 g (60.5%); and those of the HFD were: 510 kcal/100 g; protein, 23.6 g (18.3%); lipids, 34.9 g (60.9%); and carbohydrates, 25.9 g (20.8%). The fatty acid composition of the HFD was 41.2% saturated fatty acids and 58.8% unsaturated fatty acids (from mono- and polyunsaturated fatty acids, the latter of which includes phospholipid components, such as linoleic acid, linolenic acid, and arachidonic acid).

### Outline of the experiment

Rats were acclimated for one week from 3 weeks old and divided into the ND (*n* = 6) and HFD (n = 6) groups. They ingested the test diets for 11 weeks. Body weights and abdominal circumferences were measured, blood was collected from the heart, and the liver was excised at 15 weeks old. After the extraction of Mt, respiratory activity and substrate oxygen consumption in the liver were analyzed. Regarding Mt, sub-mitochondrial particles (SMP) were prepared to isolate the electron transport chain, and O·_2_^−^ production was measured. Mt and SMP were subjected to membrane fluidity measurements. Samples were stored at − 80°C until analyzed.

### Blood chemistry

The blood chemistry items examined were total cholesterol (T-ch), triglycerides (TG), HDL cholesterol (HDL), LDL cholesterol (LDL), free fatty acids (FFA), glycated hemoglobin A1c (HbA1c), and glycoalbumin (GA). The augmentation index (AI) was calculated using the formula: (T-ch – HDL)/HDL [18–20].

### Extraction of liver Mt

Mt were extracted as follows [21]: The liver was homogenized and centrifuged at 600×*g* for 10 min. The supernatant was then centrifuged twice at 8000×*g* for 10 min and precipitated Mt were collected. The Mt protein level was adjusted to 15–30 mg/mL using the Bradford method [22].

### Measurement of Mt respiratory activity and substrate oxygen consumption

Mt respiratory activity was measured using Clark’s oxygen electrode (the liquid-phase oxygen monitoring system Oxygraph, Hansatech Instruments). Mt were subjected to measurements of the respiratory control ratio (RCR), ADP/oxygen ratio (P/O ratio), and oxygen consumption with succinic acid as a substrate.

**Fig. 1:**
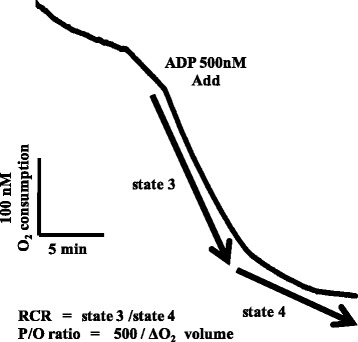
A typical example of oxygen consumption in mitochondria. Figure 1 shows the transition of Mt oxygen consumption using an oxygen electrode. A sample was added to ADP, and phosphorylation (State 3) and ATP synthesis (State 4) were measured. Based on the values obtained, the respiratory control ratio (RCR) and ADP/oxygen ratio (P/O ratio) were calculated

RCR was measured as follows [23–26]: After confirming endogenous respiration (state 1) by incubating 50 μL of Mt in 875 μL of buffer (0.25 M sucrose, 0.1 M HEPES, 0.1 M EDTA, 20 mM KCl, 2 mM MgCl_2_, and 3 mM KHPO_4_, pH 7.4) at 25°C, 30 μL of glutamic acid (15 mM) and 30 μL of malic acid (15 mM) were added, and phosphorylation with the addition of 5 μL of ADP (0.5 μM) (state 3) and ATP synthesis (state 4) were then measured. RCR was calculated from oxygen consumption in states 3 and 4 using the formula: state 3/state 4 (Fig. 1) [27]. The P/O ratio reflects the ATP-synthesizing activity of Mt, and was calculated using the following formula: Amount of ADP added/oxygen consumption in the presence of ADP (Fig. 1) [28].

Substrate oxygen consumption was measured as follows: After incubating 50 μL of Mt in 930 μL of buffer (0.25 M sucrose, 0.1 M HEPES, 0.1 M EDTA, 20 mM KCl, and 2 mM MgCl_2_, pH 7.4) at 25°C, 10 μL of succinic acid (5 mM) was added, and after confirming substrate oxygen consumption, 10 μL of antimycin A (1 μM), which is a specific inhibitor of the electron transport chain, was added to stop substrate oxygen consumption. The respiratory activity and substrate oxygen consumption of Mt were calculated with corrections of the maximum slope with the protein level.

### Preparation of SMP

SMP were prepared as follows [29, 30]: Mt were sonicated (Bioruptor, Cosmo Bio Co., Ltd.) followed by ultrasonication at Level 5, high, interval ON, 1.0 s; OFF, 0.5 s (75 s in total). The sonicated sample was subjected to ultracentrifugation at 27,000×*g* for 10 min, the supernatant was centrifuged 3 times at 77,000×*g* for 60 min, and precipitated SMP (10–20 mg/mL) were subjected to analyses.

### Measurement of O·_2_^−^

O·_2_^−^ production by SMP was measured using the slightly modified adrenaline method [29, 30] and a dual-wavelength recording spectrophotometer. The measurement conditions were set at a wavelength of 485–575 nm and 37°C. After the incubation of 25 μL of SMP combined with 900 μL of buffer (0.25 M sucrose, 0.1 M HEPES, and 0.1 M EDTA, pH 7.4), 15 μL of succinic acid (7.5 mM), 10 μL of antimycin A (1 μM), 10 μL of rotenone (1 μM), and 10 μL of adrenaline (1 mM) for 5 min, 30 μL of NADH (0.9 mM) were added, and absorbance was measured to assess the amount of O·_2_^−^ produced. It was calculated using the following formula: Adrenochrome production (min)/ millimolar extinction coefficient (2.96 mM^− 1^• cm^− 1^) × protein content in the sample (mg). The measured value was corrected by subtracting the value measured in the presence of the O·_2_^−^-scavenging enzyme superoxide dismutase (SOD; 1070 units).

### Analysis of membrane fluidity

**Fig. 2:**
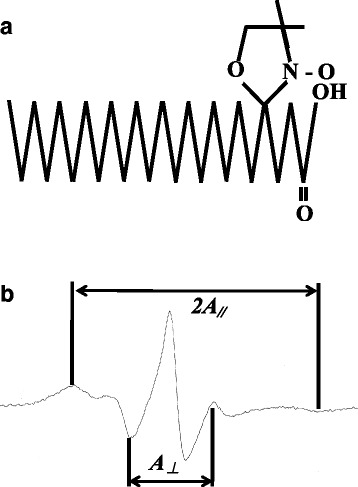
Molecular structure (**a**) and ESR spectrum (**b**) of 5-nitroxyl stearate (5-NS). **a** 5-Nitroxyl Stearate (5-NS) was used as a labeling agent. **b** The flowability of the Mt film was measured by spin labeling using an electron spin resonance apparatus and calculating the order parameter S, which is film fluidity, from the spectrum obtained

Mt membrane fluidity was measured using the spin label method [31] and an electron spin resonance device (Electron Spin Resonance, JEOL Ltd.). Using 5-nitroxyl stearate (5-NS, Fig. 2A) as the labeling agent, membrane fluidity was measured based on the binding of the nitroxide group to the alkyl chain of the phospholipid head on the external surface of the membrane. The sample was SMP, namely, the inner membrane of Mt prepared by isolating the outer membrane of Mt and the electron transport chain. In order to measure membrane fluidity, 0.6 μg of 5-NS was vaporized with nitrogen gas in a glass tube, combined with 300 μL of Mt (4 mg/mL), and then mixed for 30 min. After mixing, the sample was centrifuged at 8000×*g* for 10 min and the precipitate was subjected to measurements using ESR. ESR measurement conditions were: Sweep width, ±5.0 mT; sweep time, 3.5 min; gain, 5.0 or 6.3 × 100; modulation width, 2.0 × 0.1 mT; time constant, 0.3 s; center field, 337 mT; power, 6 mW; frequency, 9.2 GHz; and the spectrum was measured (Fig. 2B). Based on the measured spectrum, the order parameter representing membrane fluidity, S (refer to the formula below [*A*_*//*_*, A*_*⊥*_: distance of the ESR spectrum of the spin label, *A*_*zz*_*, A*_*xx*_*, A*_*yy*_: x-, y-, and z-axes of the principal axis of tensor]), was calculated and the principal axis of tensor was set at the principal value of 5-NS: *A*_*zz*_ = 6.3, *A*_*xx*_ = 5.8, *A*_*yy*_ = 33.6 (Fig. 2B). Fluidity decreases as the order parameter, S, becomes closer to 1, and increases as S becomes closer to 0.

$$ S={A}_{//}-{A}_{\perp }/{A}_{zz}\hbox{--} 1/2\left({A}_{xx}+{A}_{yy}\right) $$

### Statistical analysis

Results are presented as the mean ± standard error (mean ± SE). Changes in body weight were subjected to a two-way layout analysis of variance with 2 factors: the dietary condition and time-course changes, and the dietary condition was compared using the unpaired Student’s *t*-test, setting the significance level at 5%.

## Results

### Body composition and food intake

**Fig. 3:**
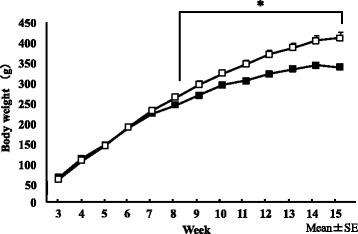
Changes in body weight in ND (■) and HFD (□) groups. We conducted a two-way ANOVA of dietary conditions and time-course variance to change body weight and set the significance level to 5%. Body weight gain was significantly higher in the HFD group than in the ND group from the 8th week. Error bars represent the standard deviation

Regarding changes in body weight during the dietary period between 3 and 15 weeks old (Fig. 3), body weight was significantly higher from week 8 in the HFD group than in the ND group (*p* < 0.05), and body weights were 412.7 ± 13.2 and 339.5 ± 5.3 g, respectively, at the time of autopsy. Regarding body composition at 15 weeks old (Table 1), the liver wet weight was not significantly different between the dietary conditions examined. Abdominal circumference was significantly higher in the HFD group than in the ND group (*p* < 0.01), and the liver/body weight ratio was higher in the ND group than in the HFD group (p < 0.01).

**Table 1:** Physical characteristics of ND and HFD groups

	ND	HFD
Abdominal circumference (cm)	18.8 ± 0.3	21.7 ± 0.3*
Liver wet weight (g)	9.8 ± 0.2	10.2 ± 0.4
Liver/Weight ratio	2.89 ± 0.03*	2.47 ± 0.06

Mean ± SE

Values are after the consumption of each diet for 11 weeks measured at dissection (n = 6). The HFD group had a higher abdominal circumference than the ND group, with a lower liver/body weight ratio (*p < 0.05)

Mean daily food intakes per rat at 15 weeks old were 14.2 and 16.4 g in the HFD and ND groups, respectively, and energy intakes were 73.1 and 59.2 kcal, respectively.

### Blood chemistry

The results of blood chemistry examinations (Table 2) showed no significant differences in T-ch, HDL, LDL, TG, FFA, HbA1c, or GA between the 2 dietary groups, whereas AI was significantly higher in the HFD group than in the ND group (*p* < 0.05).

**Table 2:** Blood profile of ND and HFD groups

	ND	HFD
Total cholesterol (mg/dl)	55.8 ± 5.9	66.2 ± 4.9
HDL cholesterol (mg/dl)	25.8 ± 1.4	24.0 ± 0.9
LDL cholesterol (mg/dl)	7.0 ± 2.9	14.7 ± 6.3
Triglyceride (mg/dl)	104.0 ± 12.6	85.8 ± 9.5
Free fatty acid (mEq/l)	1.1 ± 0.1	1.0 ± 0.1
Glycated hemoglobin A1c (%)	3.7 ± 0.1	3.7 ± 0.1
Glycoalbmin(%)	3.8 ± 0.2	3.8 ± 0.1
Augmentation index	1.1 ± 0.1	1.8 ± 0.2*

Mean ± SE

Values are blood test results after ingesting each diet for 11 weeks (n = 6)

Although the HFD group showed no significant changes in carbohydrate and lipid test items from the ND group, the arterial stiffness index was high (*p < 0.05)

### Mt respiratory activity and substrate oxygen consumption

**Fig. 4:**
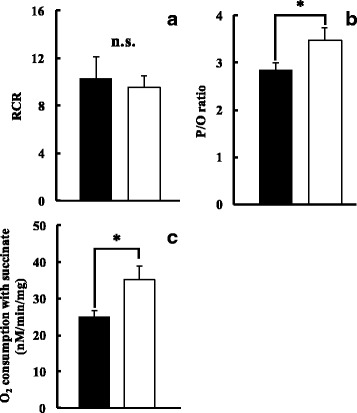
Respiratory control ratio (RCR,**a**), P/O ratio (**b**), and O_2_ consumption with succinate (**c**) in ND (■) and HFD (□) groups. Values are changes in Mt respiratory activity after ingesting each meal for 11 weeks (*n* = 6). The *t*-test was used to change dietary conditions (*p < 0.05, n.s.: no significance). The P/O ratio and oxygen consumption of succinate were higher in the HFD group than in the ND group. Error bars represent the standard deviation

RCR and the P/O ratio representing Mt respiratory activity and substrate oxygen consumption with succinic acid were compared in Fig. 4. RCR were 9.55 ± 1.00 and 10.31 ± 1.83 in the HFD and ND groups, respectively, and were not significantly different. P/O ratios were 3.48 ± 0.25 and 2.84 ± 0.16 in the HFD and ND groups, respectively, and was significantly in the HFD group (*p* < 0.05). Substrate oxygen consumption in the HFD and ND groups were 35.35 ± 3.58 and 24.83 ± 1.82 nM/min/mg, respectively, and was significantly higher in the HFD group (*p* < 0.05).

### O·_2_^−^ production

**Fig. 5:**
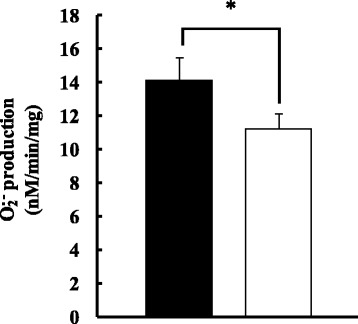
O·_2_^−^ production by the adrenaline method in ND (■) and HFD (□) groups. Values are changes in Mt O·_2_^−^ production after ingesting each diet for 11 weeks (n = 6). The *t*-test was used to change dietary conditions (*p < 0.05). O·_2_^−^ values were lower in the HFD group than in the ND group. Error bars represent the standard deviation

O·_2_^−^ production by SMP (Fig. 5) was 14.12 ± 1.32 nM/min/mg in the ND group and 11.19 ± 0.89 nM/min/mg in the HFD group, and was significantly lower in the HFD group (*p* < 0.05).

### Membrane fluidity

**Fig. 6:**
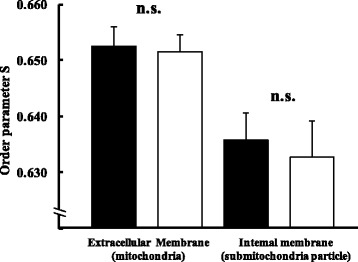
Membrane fluidity of the cell membrane in ND (■) and HFD (□) groups. Values are changes in Mt membrane fluidity after ingesting each diet for 11 weeks (n = 6). The *t*-test was used for changes in dietary conditions (no significance). The intake of a high fat diet for 11 weeks did not change Mt membrane fluidity. Error bars represent the standard deviation

When the fluidities of the outer and inner membranes of Mt were measured (Fig. 6), values for the outer membrane were 0.653 ± 0.003 in the ND group and 0.652 ± 0.003 in the HFD group, while those for the inner membrane were 0.636 ± 0.005 and 0.633 ± 0.007, respectively, and were not significantly different between the two dietary conditions.

## Discussion

The present study investigated the influence of diet-induced obesity on liver Mt O·_2_^−^ production and membrane fluidity in rats that had consumed HFD for 11 weeks. Energy metabolism was higher and O·_2_^−^ production was lower in HFD-fed rats than in ND-fed rats. No significant changes were observed in Mt membrane fluidity.

The period from 4 to 15 weeks old, during which rats were fed the HFD, accounts for suckling over puberty, which corresponds to adolescence [32]. Body weights (Fig. 3) and abdominal circumferences (Table 1) were markedly higher in the HFD group than in the ND group, and AI was higher in the HFD group (Table 2). Regarding the respiratory activity of liver Mt, RCR was 3 or higher, and the P/O ratio was 2.6 or higher [33], showing that it was possible to extract Mt in a favorable state under both conditions. The P/O ratio, reflecting the amount of ATP synthesized by Mt, and substrate oxygen consumption were higher in the HFD group than in the ND group (Fig. 4). In rats fed a HFD, fatty acid metabolism was found to be promoted, energy metabolism increased [34], and citrate synthase activity was enhanced [35]. The amount of ATP synthesized increases in the liver in the compensatory phase [36], and the ingestion of a HFD increases the total amount of Mt [35]. RCR and the P/O ratio reflect the state and impermeability of the Mt inner membrane, substrate oxidization, and conjugation with oxidative phosphorylation. In HFD-fed rats, Mt functions, such as ATP synthesis and oxidative phosphorylation, were promoted to metabolize the fatty acids ingested.

O·_2_^−^ production was measured in samples after removing the scavenging activity of Mn-SOD by ultrasonication using the adrenaline method. In the adrenaline method, adrenaline is oxidized by O·_2_^−^, and O·_2_^−^ is quantitated based on one molecule of O·_2_^−^ producing one molecule of adrenochrome. In Mt, 3–5% of oxygen becomes O·_2_^−^ during ATP synthesis in the electron transport chain [13, 14] and is scavenged by the SOD, Mn-SOD [37]. O·_2_^−^ production was lower in the HFD group than in the ND group (Fig. 5). In elderly rats fed a HFD, hydrogen peroxide production in the liver increased, RCR decreased [17], and hydrogen peroxide production in skeletal muscle increased [8]. In obese individuals, the amount of Mt decreased, thereby reducing Mt functions, such as oxygen consumption [38, 39]. We also previously demonstrated that oxidative stress may lead to cell damage in the skeletal muscle of diet-induced obese rats [40]. Regarding antioxidant capacity, the transcription level of antioxidative enzyme-related genes decreased in rats fed a HFD [41]. The increase in oxygen consumption and decrease in O·_2_^−^ production accompanying the promotion of energy metabolism indicate a reduced antioxidant capacity, such as Mn-SOD expression [8]. In the present study, the ingestion of the HFD increased ATP synthesis and reduced O·_2_^−^ production due to the promotion of fatty acid metabolism in the liver, suggesting that a reduction in antioxidant capacity occurred in the body. The genomic DNA of Mt is not protected by histones and is readily impaired by radicals. The respiratory chain, which plays a central role in energy metabolism, is located in close proximity, making it more susceptible to damage by ROS than in other cells. In a state of increased ROS production, such as exercise, exposure to ROS increases due to a reduction in the antioxidant capacity in the liver, which increases the possibility of cell damage by oxidative stress.

Moderate O·_2_^−^ production is beneficial for up-regulating the infection-protective immune system and signal transmission for apoptosis in the body [9–11, 42]. A previous study reported that ROS production by Mt increases the phagocytic and migration abilities of macrophages [43]. Taking the function of ROS as a signaling molecule into account, the ingestion of a HFD from the juvenile period may impair liver Mt and have a negative influence due to reductions in ROS production in Mt

In the present study, rats were fed lard with a high content of polyunsaturated fatty acids, which are cell membrane components. Many previous studies have employed aging model rats, dietary restrictions, and measurements of membrane fluidity in white blood cells [31, 44–46]. The spin label method used in the present study employed the labeling agent, 5-NS, and outer membrane fluidity was measured based on the nitroxide group binding to the alkyl chain of the phospholipid head on the external membrane surface. The relationship between O·_2_^−^ generation and membrane fluidity in Mt has not yet been examined. Therefore, we attempted to investigate this relationship in the livers of rats fed a HFD. Lipids account for 25–30% of Mt membrane components, and polyunsaturated fatty acids and cholesterol are abundant [47]. We hypothesized that diet-induced obesity promotes structural changes in membrane phospholipids localized in the electron transport chain and increases O·_2_^−^ leakage; however, no diet-induced change was noted in the fluidity of the Mt outer membrane or electron transport chain isolated by ultrasonication (Fig. 6). Although previous studies reported that membrane fluidity was altered with changes in the contents of cholesterol and phospholipids [15, 16], these findings were not consistent with the present study. The ingestion of a HFD has been shown to promote fatty acid oxidation due to changes in the fat composition of Mt and impaired oxidative phosphorylation [17]. Therefore, the promotion of lipid metabolism in the liver may have resulted in a decrease in lipid infiltration in the liver and had no influence on the Mt membrane.

Regarding the limitations of the present study, we isolated and analyzed the electron transport chain from Mt, but did not quantify O·_2_^−^ in Mt after substrate-permeable cell processing. Future studies are needed in order to investigate this and examine the impact of the duration of a HFD on Mt and O·_2_^−^. Another aspect that warrants further study is the impact of an exercise protocol on antioxidant capacity and O·_2_^−^ production.

## Conclusion

The purpose of the present study was to evaluate the impact of HFD-induced obesity on Mt O·_2_^−^ production in the liver and assess changes in Mt membrane fluidity. We found that a HFD increased Mt respiratory activity and reduced O·_2_^−^ production, but did not change membrane fluidity. Although O·_2_^−^ damages cells as an oxidative stress, ROS and SOD are essential signaling molecules in the body. The present results suggest that the continuous ingestion of a HFD impairs Mt and induces disease development.

## Abbreviations

5-NS: 5-nitroxyl stearate
AI: augmentation index
FFA: free fatty acids
GA: glycoalbumin
HbA1c: glycated hemoglobin A1c
HDL: HDL cholesterol
HFD: high-fat diet
LDL: LDL cholesterol
Mt: mitochondrial
ND: normal diet
O·_2_^−^: superoxide anion
P/O ratio: ADP/oxygen ratio
RCR: respiratory control ratio
ROS: reactive oxygen species
SMP: submitochondrial particles
SOD: superoxide dismutase
T-ch: total cholesterol
TG: triglycerides
